# 

**Published:** 1894-12

**Authors:** 


### CONTENTS FOR DECEMBER, 1894.

ORIGINAL COMMUNICATION. PAGE.

A Criticism of the "Germ Theory of Disease," Based on the Baconian Method. By Lawson Tait, F. R. C. S. .... 257
Hydrotherapy in Appendicitis. By George E. Clark, M. D. .... 282
Some Points in the Etiology, Pathology and Indicated Treatment of Diphtheria. By A. A. Young, M. D. .... 288

REPORTS OF SOCIETIES.

The Eighth Periodical International Ophthalmological Congress .... 295

CLINICAL REPORT.

An Interesting Case of Hysteria. By James Stafford, M. D. .... 300

PROGRESS IN MEDICAL SCIENCE.

Surgery .... 301
Therapeutic Notes .... 302

EDITORIAL.

The Mississippi Valley Medical Association .... 303
State Examinations for License, New York .... 306
Topics of the Month .... 307

Personal .... 309
Obituary .... 310
Book Reviews .... 311
Miscellany .... 318

